# Therapeutic Alliance in Web-Based Treatment for Eating Disorders: Secondary Analysis of a Randomized Controlled Trial

**DOI:** 10.2196/33813

**Published:** 2022-06-30

**Authors:** Claudia Stoeten, Hein Arnoud de Haan, Marloes Gerda Postel, Marjolein Brusse-Keizer, Elke Daniëlle ter Huurne

**Affiliations:** 1 Tactus Addiction Care Deventer Netherlands; 2 Mediant Hengelo Netherlands; 3 Nijmegen Institute for Scientist-Practitioners in Addiction (NISPA) Nijmegen Netherlands; 4 Saxion University of Applied Sciences Enschede Netherlands; 5 Medical School Twente Medisch Spectrum Twente Enschede Netherlands

**Keywords:** therapeutic alliance, TA, treatment completion, cognitive behavioral therapy, CBT, web-CBT, eating disorders

## Abstract

**Background:**

In face-to-face therapy for eating disorders, therapeutic alliance (TA) is an important predictor of symptom reduction and treatment completion. To date, however, little is known about TA during web-based cognitive behavioral therapy (web-CBT) and its association with symptom reduction, treatment completion, and the perspectives of patients versus therapists.

**Objective:**

This study aimed to investigate TA ratings measured at interim and after treatment, separately for patients and therapists; the degree of agreement between therapists and patients (treatment completers and noncompleters) for TA ratings; and associations between patient and therapist TA ratings and both eating disorder pathology and treatment completion.

**Methods:**

A secondary analysis was performed on randomized controlled trial data of a web-CBT intervention for eating disorders. Participants were 170 females with bulimia nervosa (n=33), binge eating disorder (n=68), or eating disorder not otherwise specified (n=69); the mean age was 39.6 (SD 11.5) years. TA was operationalized using the Helping Alliance Questionnaire (HAQ). Paired t tests were conducted to assess the change in TA from interim to after treatment. Intraclass correlations were calculated to determine cross-informant agreement with regard to HAQ scores between patients and therapists. A total of 2 stepwise regressive procedures (at interim and after treatment) were used to examine which HAQ scores predicted eating disorder pathology and therapy completion.

**Results:**

For treatment completers (128/170, 75.3%), the HAQ-total scores and HAQ-Helpfulness scores for both patients and therapists improved significantly from interim to post treatment. For noncompleters (42/170, 24.7%), all HAQ scores decreased significantly. For all HAQ scales, the agreement between patients and therapists was poor. However, the agreement was slightly better after treatment than at interim. Higher patient scores on the helpfulness subscale of the HAQ at interim and after treatment were associated with less eating disorder psychopathology. A positive association was found between the HAQ-total patient scores at interim and treatment completion. Finally, posttreatment HAQ-total patient scores and posttreatment HAQ-Helpfulness scores of therapists were positively associated with treatment completion.

**Conclusions:**

Our study showed that TA in web-CBT is predictive of eating disorder pathology and treatment completion. Of particular importance is patients’ confidence in their abilities as measured with the HAQ-Helpfulness subscale when predicting posttreatment eating disorder pathology and treatment completion.

## Introduction

### Background

Eating disorders (EDs) are related to serious physical, psychological, and social consequences and are characterized by a chronic character and high treatment costs [1,2]. However, many patients have EDs for years before receiving treatment [3,4]. Access to face-to-face treatment of ED is often limited because of personal barriers, such as feelings of shame and fear of stigmatization, and intervention-related barriers, such as costs, geographic distance, and lack of availability [5-9]. Web-based alternatives, which may encompass website- and mobile app–based treatment programs, might show promise. Web-based treatment was shown to be effective in reducing ED psychopathology [10-22], and it can improve access to ED treatment compared with face-to-face treatment [15,17,19]. Web-based treatment provides the added advantages of approachability, relative anonymity, and widespread 24-hour access, which are considered important benefits for patients with ED [15].

One particularly important facet of face-to-face treatment is the therapeutic alliance (TA) between therapists and patients [23,24]. Although there are various ways to define the concept of TA [23], all definitions have in common that TA can best be characterized by the degree of agreement between a therapist and a patient concerning the goals and tasks of the treatment and suggest the presence of an affective bond [23-26].

TA was shown to be predictive of treatment completion and outcomes in the general population [27]. The quality of TA was also shown to be predictive of treatment completion and outcomes in face-to-face ED treatment [28-30]. However, the predictive value of TA for treatment outcomes in patients with ED varies between studies and between patient groups [28]. More specifically, the predictive value of TA for treatment outcome is less obvious for patients with bulimia nervosa (BN) than for patients with anorexia nervosa [28]. Overall, the predictive value of TA for treatment outcomes is associated with small to medium effect sizes [30].

With regard to web-based treatment in the general population, multiple studies have demonstrated that the strength of the TA during treatment can be improved without face-to-face contact with a therapist [31-37]. However, compared with face-to-face treatment, much less is known about important predictors in the development of TA in the context of web-based treatment [31,38]. Studies focusing on TA in web-based treatment are often methodologically inferior to those focusing on face-to-face treatment [31,38].

Few studies have been conducted on the role of TA with regard to treatment outcomes and adherence in web-based treatment for ED [11,38]. It was found that TA was rated high over the course of ED treatment [39,40]. It was also found that higher TA ratings were associated with better treatment outcomes [25,27,40]. Furthermore, some evidence indicates that the extent of TA during web-based treatment of ED is positively associated with treatment adherence [11,29,41].

### Objectives

Concerning the effects of TA on treatment effectiveness, it is also important to emphasize the degree of agreement between the therapist and patient perspectives [42,43]. In the general population, it has been shown that convergent patient-therapist ratings over the course of treatment predict a better treatment outcome [42,43]. It was also found that, for face-to-face treatment in the general population, therapist ratings of the TA are not as predictive of treatment outcomes as the TA ratings provided by patients [43].

This study focused on web-based cognitive behavioral therapy (web-CBT) for female patients with ED and aimed to investigate (1) TA ratings measured at interim and after treatment, separately for patients (treatment completers and noncompleters) and therapists; (2) the degree of agreement between therapists and patients (treatment completers and noncompleters) for TA ratings; and (3) associations between patient and therapist TA ratings and both ED pathology and therapy completion. We hypothesized that the TA would increase from interim to post treatment for both patients and therapists and that there would be stronger agreement between therapists and patients who completed treatment than between therapists and patients who did not complete treatment. Furthermore, we hypothesized that TA ratings provided by patients and therapists are predictors of eating disorder pathology, particularly after treatment. Finally, we hypothesized that TA ratings would be positively associated with treatment completion.

## Methods

### Study Design

A secondary analysis was conducted on the data from a randomized controlled trial (RCT) investigating a web-CBT intervention for EDs. The study design, procedures, and results of the RCT are described in detail elsewhere [19,41,44,45]. Recruitment for the RCT was conducted from March 2011 to December 2013. Information on the study was disseminated through announcements on ED-related websites, forums, and newspaper advertisements.

### Ethics Approval

All participants provided written informed consent, and the study was approved by the Medical Ethics Committee of the Medical Spectrum Twente (NL31717.044.010, P10-31) and registered in the Netherlands Trial Registry (NTR2415).

An RCT [19,44] compared a web-CBT intervention group to a waiting list control group. Participants were stratified by ED type (BN, binge eating disorder [BED], or eating disorder not otherwise specified [EDNOS]). Participants in the intervention group started web-CBT immediately, whereas those in the control group had to wait 15 weeks after randomization. Both completers and noncompleters completed the questionnaire. Outcomes were measured before, during, and after web-CBT and at 3-, 6-, and 12-month follow-up.

For the current analysis, measurements at the interim (after the first part of treatment) and after treatment were used. As this study did not focus on the efficacy of the treatment but on the interim and posttreatment measurements of the TA, the data from the intervention phase of the study of both the initial intervention and control groups were merged.

### Participants

The participants of this study were female patients with a Diagnostic and Statistical Manual of Mental Disorders, Fourth Edition (DSM-IV) diagnosis of BN, BED, or EDNOS who completed the first part of the web-based CBT and completed the interim questionnaire. In addition to the DSM-IV classification, the inclusion criteria for the RCT were (1) age ≥18 years, (2) access to the internet, (3) fluent in Dutch, (4) referral from a general practitioner, and (5) to be within 85% of the target weight established by the table of height and weight limits of MINI-Plus [46,47]. Exclusion criteria were as follows: (1) suicidal ideation, (2) receiving psychological or pharmaceutical treatment for any ED within the past 6 months, (3) pregnancy, and (4) expected absence of 4 weeks or longer during the treatment period of 15 weeks.

### Intervention

The web-based treatment program, Etendebaas (English translation: “Look at your eating”), included a structured 15-week web-CBT that was designed within a secure web-based application [19,44]. The treatment program consisted of 2 parts and included 16 treatment modules, with at least 21 scheduled asynchronous contact moments and 10 homework assignments. The first part aimed to analyze participants’ ED attitudes and behaviors, whereas the second part focused on behavioral changes. All treatment modules were completed by the patients in a fixed order, and it was not possible to skip a module.

CBT [48-50] and motivational interviewing [51,52] were the fundamental elements of the intervention, which included techniques such as psychoeducation, self-monitoring through daily diary entries, thought restructuring, problem-solving, and relapse prevention. In their personal files, patients could read and respond to the therapist’s messages and complete homework assignments. The treatment protocol prescribed regular contact between patients and their therapists, with therapists responding to the patients’ messages and assignments within 3 working days.

A total of 17 therapists carried out web-based treatments, including 2 male therapists and 15 female therapists. Therapists had either a bachelor’s degree in nursing or social work or a master’s degree in psychology and received specific training for web-based treatment. A comprehensive manual was available, which included a detailed description of all treatment modules and safety protocols. The treatment also included web-based coaches and support from a multidisciplinary team (psychologists, psychotherapists, addiction medicine physicians, psychiatrists, and dieticians) who were available for consultation. The participating therapists did not have knowledge of the TA scores of the patients and did not receive any instructions regarding investing in improving the TA. However, within the regular web-based treatment protocol, the core task of a therapist is to build and maintain the TA.

### Measures

#### Therapeutic Alliance

TA was measured using the Dutch patient and therapist version of the Helping Alliance Questionnaire (HAQ) [53,54]. The HAQ is a self-report questionnaire that measures the strength of a therapeutic patient-therapist alliance [55]. The therapist’s version was derived from the patient’s version and was compatible. The Dutch version of the HAQ has 11 items scored on a 5-point Likert scale (1=totally disagree, 2=disagree, 3=neutral, 4=agree, and 5=totally agree) [54]. The HAQ contains two subscales: (1) cooperation (5 items), reflecting the perception of the patient on the cooperation with a care provider or vice versa, and (2) helpfulness (6 items), which reflects a patient’s or therapist’s confidence in their own capacity to improve the situation. The HAQ-total score was determined as the sum of the subscale scores. This study found the patient version of the HAQ to be internally reliable at the interim measurement: Cronbach α of .81 for the cooperation subscale, Cronbach α of .81 for the helpfulness subscale, and Cronbach α of .87 for the total HAQ score. The therapist version of the HAQ was also internally reliable: Cronbach α of .77 for the cooperation subscale, Cronbach α of .78 for the helpfulness subscale, and Cronbach α of .87 for the total HAQ score.

#### Eating Disorder Psychopathology

Changes in the clinical severity of ED psychopathology were measured using the total score of the Eating Disorder Examination Questionnaire (EDE-Q) [56]. The EDE-Q is a widely used validated self-report scale based on Eating Disorder Examination interviews. The instrument focuses on the previous 28 days to assess important behavioral and attitude aspects of ED and the severity of ED psychopathology. The EDE-Q consists of 36 items, with four subscales (restraint, eating concern, shape concern, and weight concern). Items are scored on a 7-point Likert scale (range 0=not one single day–6=every day), with a higher score reflecting more psychopathology. Subscale scores were obtained by averaging the items for each subscale, whereas the total EDE-Q score was obtained by summing the subscale scores. Previous research indicates that the EDE-Q demonstrates acceptable internal consistency (Cronbach α ranging from .77 to .84) [57-59].

#### Treatment Completion

Participants were considered completers when they (1) had attended all 16 treatment modules with at least 21 contact moments with their personal therapist, (2) completed all 10 homework assignments, and (3) completed the at-interim and posttreatment questionnaires. Participants who stopped the treatment program before the completion of all treatment modules and completed the at-interim and posttreatment questionnaires were considered noncompleters. Therefore, treatment completion was operationalized using a dichotomous measure (yes or no).

### Statistical Analysis

All analyses were conducted using SPSS for Windows (version 21; IBM Corp) [60]. Continuous variables were summarized using the mean with the associated SD or as the median with the associated IQR for normally and nonnormally distributed data, respectively. Categorical variables were summarized as frequencies with corresponding percentages. Sum scores were computed for the HAQ-total score and separately for the cooperation and helpfulness subscales, both at interim and after treatment, and separately for patients and therapists.

Differences in demographic characteristics between completers and noncompleters were analyzed using independent 2-tailed *t* tests for continuous normally distributed data and Wilcoxon rank-sum tests for continuous nonnormally distributed data. Differences in categorical variables were analyzed using chi-square or Fisher exact tests (as appropriate).

Paired *t* tests were conducted to assess the change in TA ratings from the interim to the end of treatment for both therapists and patients. In the analyses, we stratified for completers and noncompleters because we expected an opposite pattern in TA ratings from interim to post treatment. Cohen *d*=(µ_1_−µ_2_)/σ_1,2_ was calculated to determine the effect sizes for significant findings. Cohen defines *d* scores of 0.2, 0.5, and 0.8 as small, medium, and large effects, respectively [61]. An intraclass correlation coefficient (ICC) analysis was conducted to determine cross-informant agreement between patients (separately for completers and noncompleters) and therapists in TA ratings, both at interim and post treatment. To determine the strength of agreement, the guidelines drafted by Koo and Li [62] were used (<0.50: poor, between 0.50 and 0.75: moderate, between 0.75 and 0.90: good, and >0.90: excellent) [62].

Next, we examined whether the HAQ scores for patients and therapists were related to ED pathology by creating two linear regression models: one at interim and one post treatment. To do this, TA ratings of therapists and patients (completers and noncompleters combined) were first analyzed separately in univariate linear regression models. We merged the data of completers and noncompleters. The choice to combine completers and noncompleters was based on the following arguments: (1) this would increase statistical power; (2) this would provide fairer insights, as TA ratings of completers were expected to be overly positive specifically because these patients completed the treatment, whereas TA ratings of noncompleters may have been more critical; and (3) by including both groups, the range of ED pathology included in the analyses was broader, increasing the ecological validity of the results. Rating scores that were sufficiently related (*P*≤.15) in these univariate analyses were entered into a multiple linear regression model. Owing to multicollinearity between the TA subscales and total scale within the patient or therapist groups, we entered the total scale or subscales with the highest explained variance (*R*^2^) into the multiple linear regression model. Nonsignificant variables were removed individually until the explained variance deteriorated significantly.

To assess whether TA ratings were related to treatment completion, we constructed two logistic regression models: one at interim and one post treatment. These models were constructed identically to the construction of the previously described multiple regression models. Owing to multicollinearity between the TA subscales and the total scale for the patient or therapist groups at each time point, we entered the scale(s) that produced the best model fit (−2 log likelihood). Nonsignificant variables were removed one by one until the −2 log likelihood deteriorated significantly. Nagelkerke *R*^2^ was used to estimate the pseudoproportion of the variance. Two-sided significance levels were set to 0.05 in all measurements.

## Results

### Inclusion Process

**Figure 1:**
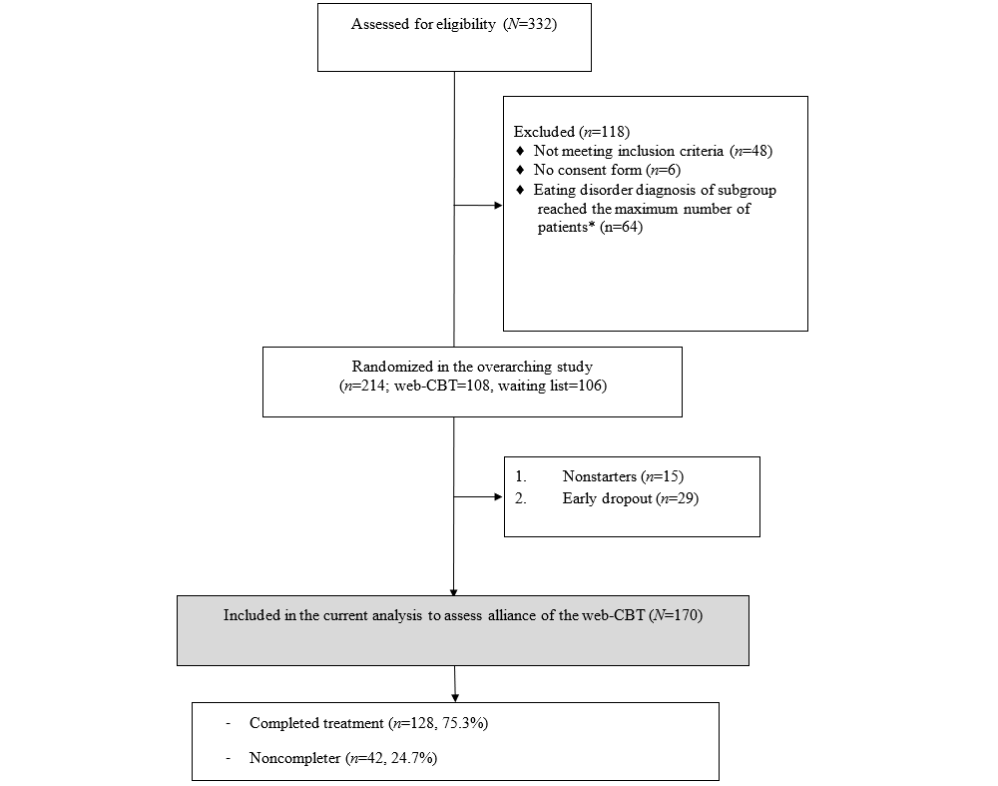
Flowchart of the inclusion process of this study. *In the underlying RCT, power analysis was used to determine how many participants could be assigned to each subgroup. The number of patients included in the binge eating disorder (BED) and eating disorder not otherwise specified (EDNOS) subgroups reached the necessary number of patients that should be recruited within the subgroup based on the sample size calculation, and the necessary number of patients for the bulimia nervosa (BN) group was not reached [19,44]. web-CBT: web-based cognitive behavioral therapy.

Figure 1 presents a flowchart of the inclusion process used in this study. A total of 214 participants were included in an earlier RCT [19,44]. Of these, 128 (59.8%) completed the treatment. Of the participants who did not complete the web-based CBT (86/214, 40.1%), 15 never started treatment (nonstarters), and 29 stopped the treatment before the end of the first part of the treatment (early dropouts). The 44 participants did not complete the interim questionnaire, so no information about their experiences with the TA was available. Therefore, these were excluded from the analysis. Of the 170 participants who were included in this study, 42 stopped the program during the second part of the web-based CBT. These participants were considered late dropouts and filled out the interim and posttreatment questionnaires, including the TA. In this study, late dropouts were defined as noncompleters, although the overall number of noncompleters in the RCT was higher (n=106), as it also included 15 nonstarters and 29 early dropouts.

### Participants

The participant characteristics are reported in Table 1. The sample included 170 women with BN (n=33), BED (n=68), or EDNOS (n=69), and a mean age of 39.6 (SD 11.5) years. Completers reported significantly higher BMI scores than noncompleters. When stratifying for BMI categories (underweight, normal weight, and overweight), no significant differences were found between these groups.

**Table 1:** Participants characteristics for completers and noncompleters.

Variable		Overall (N=170)	Completers (n=128)	Noncompleters (n=42)	*P* value	
Age (years), mean (SD)		39.6 (11.5)	40.5 (11.1)	36.6 (12.4)	.06	
BMI (kg/m^2^), mean (SD)		31.9 (6.9)	32.6 (6.9)	30 (6.8)	.04^a^	
**BMI (kg/m^2^), n (%)**						.25
	Underweight: <18.5	6 (3.5)	3 (2.3)	3 (7.1)		
	Normal weight: 18.5-25	21 (12.4)	15 (11.7)	6 (14.3)		
	Overweight: >25	143 (84.1)	110 (85.9)	33 (78.6)		
**Eating disorder, n (%)**						.67
	Bulimia nervosa	33 (19.4)	23 (18)	10 (23.8)		
	Binge eating disorder	68 (40)	53 (41.4)	15 (35.7)		
	Eating disorder not otherwise specified	69 (40.6)	52 (40.6)	17 (40.5)		
**Living situation, n** **(%)**						.31
	Alone	38 (22.4)	31 (24.2)	7 (16.7)		
	With others	132 (77.6)	97 (75.8)	35 (83.3)		
**Level of education, n (%)**						.70
	Low	18 (10.6)	15 (11.7)	3 (7.1)		
	Intermediate	59 (34.7)	44 (34.4)	15 (35.7)		
	High	93 (54.7)	69 (53.9)	24 (57.1)		
**Employment,** **n (%)**						.28
	Paid job	139 (81.8)	107 (83.6)	32 (76.2)		
	No paid job	31 (18.2)	21 (16.4)	10 (23.8)		
**Duration of eating** **disorder (years), n (%)**						.13
	1-5	24 (14.1)	14 (10.9)	10 (23.8)		
	6-10	25 (14.7)	19 (14.8)	6 (14.3)		
	11-20	53 (31.2)	39 (30.5)	14 (33.3)		
	>20	68 (40)	56 (43.8)	12 (28.6)		
**Professional treatment** **of eating disorder, n (%)**						.48
	Yes	77 (45.3)	56 (43.8)	21 (50)		
	No	93 (54.7)	72 (56.3)	21 (50)		
**Professional treatment, n (%)**						.90
	Yes	112 (65.9)	84 (65.6)	28 (66.7)		
	No	58 (34.1)	44 (34.4)	14 (33.3)		
**Medication use, n (%)**						.09
	Yes	77 (60.2)	19 (45.2)	96 (56.5)		
	No	51 (39.8)	23 (54.8)	74 (43.5)		
**Smoking, n (%)**						.66
	Yes	21 (12.4)	15 (11.7)	6 (14.3)		
	No	149 (87.6)	113 (88.3)	36 (85.7)		
**Alcohol use (experienced problematic), n (%)**						.99
	Yes	6 (4.2)	5 (4.6)	1 (2.9)		
	No	136 (95.8)	103 (95.4)	33 (97.1)		
**Drug use, n (%)**						.26
	Yes	4 (2.4)	2 (1.6)	2 (4.8)		
	No	166 (97.6)	126 (98.4)	40 (95.2)		
**Gambling, n (%)**						.99
	Yes	4 (2.4)	3 (2.3)	1 (2.4)		
	No	166 (97.6)	125 (97.7)	41 (97.6)		

^a^*P*<.05.

### TA Before and After Treatment

In Table 2, TA ratings at interim and post treatment and the difference scores between the measurements are reported separately for completers and noncompleters and from both the patient and therapist perspectives. For completers, the HAQ-total and HAQ-Helpfulness scores improved significantly from interim to the end of treatment, with effect sizes ranging from small to medium. For noncompleters, all 3 types of HAQ scores significantly decreased, with medium to large effect sizes.

**Table 2:** Helping Alliance Questionnaire scores of patients and therapists at interim and posttreatment and difference scores.

				Interim scores					Posttreatment scores					Difference scores^a^						
				N		Value, mean (SD; range)		Value, N			Value, mean (SD; range)		Value, N			Value, mean (SD)		*P* value		Cohen *d*
**Patients**																				
	**Completers**																			
		HAQ-Co^b^	128		20.6 (2.4; 13.0-25.0)		126			20.8 (2.6; 15.0-25.0)		126			0.2 (2.1)		.25		0.10	
		HAQ-HE^c^	128		23.3 (3.2; 14.0-30.0)		126			24.7 (3.1; 16.0-30.0)		126			1.4 (3.1)		<.001^d^		0.45	
		HAQ-T^e^	128		43.9 (5.0; 30.0-55.0)		126			45.5 (5.1; 32.0-55.0)		126			1.6 (4.6)		<.001^d^		0.35	
	**Noncompleters**																			
		HAQ-Co	42		18.8 (3.0; 9.0-25.0)		31			16.0 (4.9; 5.0-23.0)		31			−3.1 (4.0)		<.001^d^		−0.78	
		HAQ-HE	42		20.4 (3.8; 11.0-29.0)		31			17.8 (5.1; 8.0-28.0)		31			−2.2 (3.6)		.002^d^		−0.60	
		HAQ-T	42		39.2 (5.9; 24.0-53.0)		31			33.9 (8.2; 13.0-46.0)		31			−5.3 (6.0)		<.001^d^		−0.89	
**Therapists**																				
	**Completers**																			
		HAQ-Co	125		19.8 (1.9; 15.0-25.0)		126			20.0 (2.4; 12.0-25.0)		123			0.2 (2.2)		.42		0.07	
		HAQ-HE	125		22.6 (2.9; 11.0-28.0)		126			24.1 (3.0; 14.0-30.0)		123			1.5 (3.0)		<.001^d^		.50	
		HAQ-T	125		42.4 (4.4; 28.0-53.0)		126			44.1 (5.1; 27.0-55.0)		123			1.6 (4.7)		<.001^d^		0.35	
	**Noncompleters**																			
		HAQ-Co	42		19.3 (2.9; 13.0-25.0)		40			16.6 (3.3; 10.0-24.0)		40			−2.6 (2.8)		<.001^d^		−0.91	
		HAQ-HE	42		21.4 (3.3; 16.0-27.0)		40			18.6 (4.2; 10.0-29.0)		40			−2.6 (3.2)		<.001^d^		−0.82	
		HAQ-T	42		40.7 (5.9; 30.0-51.0)		40			35.2 (7.0; 22.0-53.0)		40			−5.2 (5.2)		<.001^d^		−0.99	

^a^Difference score=posttreatment score−interim score.

^b^HAQ-CO: Helping Alliance Questionnaire Cooperation.

^c^HAQ-HE: Helping Alliance Questionnaire Helpfulness.

^d^*P*<.05.

^e^HAQ-T: Helping Alliance Questionnaire total.

### Cross-Informant Agreement Between Patients and Therapists

The ICCs that represent agreement between therapists and patients regarding the TA (represented by the HAQ-total score and HAQ subscale scores) are presented in Table 3. Agreement between therapists and patients increased as treatment progressed. However, in general, agreement between patients and therapists was poor for both noncompleters and completers both at interim and post treatment.

**Table 3:** Intraclass correlations between therapists and patients.

		Value, *N* valid	Intraclass correlation	*P* value
**HAQ^a^-Cooperation at interim^b^**				
	Completers	125	0.09	.17
	Noncompleters	42	−0.16	.85
**HAQ-Helpfulness at interim**				
	Completers	125	0.26	.002^c^
	Noncompleters	42	−0.07	.67
**HAQ-total at interim**				
	Completers	125	0.20	.014^c^
	Noncompleters	42	−0.14	.82
**HAQ-Cooperation post treatment^d^**				
	Completers	124	0.29	.001^c^
	Noncompleters	30	0.41	.011^c^
**HAQ-Helpfulness post treatment**				
	Completers	124	0.41	<.001^c^
	Noncompleters	30	0.34	.029^c^
**HAQ-total post treatment**				
	Completers	124	0.39	<.001^c^
	Noncompleters	30	0.48	.003^b^

^a^HAQ: Helping Alliance Questionnaire.

^b^At interim, there were 3 missing participant scores.

^c^*P*<.05.

^d^Post treatment, there were 4 missing scores for completers and 12 for noncompleters.

### Associations With Treatment Outcome

For ED pathology measured with the EDE-Q, Table 4 shows the results of the univariate regression analyses at the interim. All patients’ HAQ scores at interim were found to be univariately negatively associated with the extent of ED psychopathology, as was the therapists’ HAQ-Helpfulness score at interim. The subscales showed the best explained variance; therefore, we entered these subscales and not the total HAQ scale in the initial multiple regression model. After entering the patients’ subscale scores and therapists’ scores for the helpfulness subscale and completing the stepwise regression procedure, only the HAQ-Helpfulness score at the interim of patients was significantly negatively associated with ED pathology. The final interim model with the HAQ-Helpfulness scores of patients as the sole predictor explained 9.8% (*F*_1_=17.03; *P*<.001) of the variance in eating disorder pathology.

As presented in Table 5, posttreatment HAQ scores of all patients were found to be univariately negatively associated with posttreatment ED psychopathology, as well as therapists’ HAQ-Helpfulness and HAQ-total scores after treatment. Owing to multicollinearity and the best model of fit for the subscales, all patients’ subscale scores and the therapists’ helpfulness subscale scores were entered in the initial multiple regression model. After completing the stepwise regression procedure, the HAQ-Helpfulness score of the patients remained the only predictor that was negatively associated with ED pathology after treatment. After treatment, the HAQ-Helpfulness score of patients explained 22.3% (*F*_1_=43.58; *P*<.001) of the variance in ED pathology.

**Table 4:** Univariate regression models for the at-interim association between the strength of the therapeutic alliance (Helping Alliance Questionnaire scores) and eating disorder pathology (Eating Disorder Examination Questionnaire), separately per subscale and as total score, and separately for patients and therapists.

Outcome variable at interim				Univariate coefficients (95% CI)		*P* value
**Eating Disorder Examination Questionnaire**						
	**Patients**					
		HAQ^a^-Cooperation	−0.08 (−0.14 to −0.01)		.02^b^	
		HAQ-Helpfulness	−0.10 (−0.15 to −0.05)		<.001^b^	
		HAQ-total	−0.06 (−0.09 to −0.03)		<.001^b^	
	**Therapists**					
		HAQ-Cooperation	−0.04 (−0.12 to 0.05)		.40	
		HAQ-Helpfulness	−0.06 (−0.12 to 0.001)		.06	
		HAQ-total	−0.03 (−0.07 to 0.01)		.12	

^a^HAQ: Helping Alliance Questionnaire.

^b^*P*<.05.

**Table 5:** Univariate regression models for the posttreatment association between the strength of the therapeutic alliance (Helping Alliance Questionnaire scores) and eating disorder pathology (Eating Disorder Examination Questionnaire), separately per subscale and as a total score, and separately for patients and therapists.

Outcome variable post treatment			Univariate coefficients (95% CI)	*P* value
**Eating Disorder Examination Questionnaire**				
	**Patients**			
		HAQ^a^-Cooperation	−0.09 (−0.13 to −0.04)	.001^b^
		HAQ-Helpfulness	−0.12 (−0.16 to −0.08)	<.001^b^
		HAQ-total	−0.07 (−0.09 to −0.04)	<.001^b^
	**Therapists**			
		HAQ-Cooperation	−0.03 (−0.10 to 0.04)	.39
		HAQ-Helpfulness	−0.08 (−0.12 to −0.03)	.002^b^
		HAQ-total	−0.03 (−0.06 to −0.01)	.02^b^

^a^HAQ: Helping Alliance Questionnaire.

^b^*P*<.05.

### Associations With Treatment Completion

Table 6 shows the results of the univariate logistic regression analyses at the interim of the association between HAQ scores and treatment completion. All patients’ HAQ scores at the interim measurement and the therapists’ HAQ-Helpfulness and HAQ-total score at the interim were found to be univariately associated with treatment completion. We entered patients’ HAQ-total scores and therapists’ subscale scores into the initial multiple regression model because these resulted in the best model fit. In the final multivariate model, the HAQ-total score of patients at the interim measurement remained the only significant predictor of treatment completion, explaining 18.8% (−2 log likelihood=167.10; Nagelkerke *R*^2^=0.188) of the pseudovariance in treatment completion.

Table 7 shows the results of the posttreatment univariate logistic regression analyses, focusing on HAQ scores and treatment completion. All the HAQ scores were found to be univariately positively associated with treatment completion. The HAQ-total scores of patients and the therapists’ subscales were entered into the initial model because these resulted in the best model fit. In the final multiple regression model, both the HAQ-total scores of patients (odds ratio [OR] 0.30, 95% CI 1.18-1.55; *P*≤.001) and HAQ-Helpfulness scores of therapists (OR 0.13, 95% CI 0.97-1.34; *P*=.12) were positively associated with treatment completion, explaining 59% (−2 likelihood=80.24; *R*^2^=0.59) of the pseudovariance in treatment completion.

**Table 6:** Univariate regression models at interim Helping Alliance Questionnaire scores and treatment completion.

		Odds ratio (95% CI)	*P* value
**Patients**			
	HAQ^a^-Cooperation	1.30 (1.12-1.51)	<.001^b^
	HAQ-Helpfulness	1.29 (1.15-1.46)	<.001^b^
	HAQ-total	1.18 (1.10-1.28)	<.001^b^
**Therapists**			
	HAQ-Cooperation	1.12 (0.95-1.32)	.17
	HAQ-Helpfulness	1.13 (1.01-1.27)	.03^b^
	HAQ-total	1.07 (1.00-1.15)	.05^b^

^a^HAQ: Helping Alliance Questionnaire.

^b^*P*<.05.

**Table 7:** Univariate regression models posttreatment Helping Alliance Questionnaire scores and treatment completion.

		Odds ratio (95% CI)	*P* value
**Patients**			
	HAQ^a^-Cooperation	1.58 (1.30-1.92)	<.001^b^
	HAQ-Helpfulness	1.58 (1.34-1.87)	<.001^b^
	HAQ-total	1.40 (1.23-1.58)	<.001^b^
**Therapists**			
	HAQ-Cooperation	1.54 (1.32-1.80)	<.001^b^
	HAQ-Helpfulness	1.51 (1.32-1.73)	<.001^b^
	HAQ-total	1.27 (1.17-1.37)	<.001^b^

^a^HAQ: Helping Alliance Questionnaire.

^b^*P*<.05.

## Discussion

### Principal Findings

First, in line with our expectations, it is possible to examine and detect changes in the TA of web-based CBT. Our study showed that the HAQ-total and HAQ-Helpfulness scores for completers significantly increased from interim to post treatment, whereas for noncompleters, all 3 HAQ scores significantly decreased. This shows that, in general, patients who completed treatment experienced a TA that grew stronger over time, whereas those who did not complete treatment experienced a weaker TA that decreased over time. These findings were observed for both patients and therapists and confirmed the results of previous studies [44,63].

In addition, we found that although the ICCs of agreement between patients and therapists increased from interim to posttreatment measurement, the overall agreement about the degree of TA remained relatively poor. This might partly be because of differences in perceptions between patients and therapists regarding what the TA entails. Patients are more concerned with the helpfulness of the treatment and collaboration, whereas therapists are more concerned with the performance of the client and their own confidence as therapists [64].

Patients’ interim and posttreatment HAQ scores were positively associated with treatment outcomes. The final interim model with the HAQ-Helpfulness scores of patients as the sole predictor explained 9.8% (*F*_1_=17.03; *P*<.001) of the variance in eating disorder pathology. Post treatment, the HAQ-Helpfulness score of patients explained 22.3% (*F*_1_=43.58; *P*<.001) of the variance in eating disorder pathology. This corresponds with the results of other studies [27,30,65]. Although the explained variance is not that high, meaning that there are more unknown factors influencing eating disorder pathology, it does show that patients who are confident in their own capacity to improve their situation are more likely to have better treatment outcomes. This is in line with a narrative review of web-based interventions that showed that in most studies, helpfulness-related factors were found to be positively associated with treatment outcomes in internet interventions [33,66]. In only one of the studies described in the narrative review, a positive association between cooperation-related factors and treatment outcomes was found [33]. Patients who opt for web-CBT may value cooperation with a therapist less important than patients who prefer face-to-face treatment and may prefer the relative anonymity of the internet [64]. For web-CBT for ED, we found one study that also reported a positive association between TA and treatment outcomes [65]. However, this study used a different measure to operationalize the construct of TA and did not focus on the perspective of therapists.

Finally, treatment completion is an important predictor of treatment outcome [41,66,67]. In a previous study by our laboratory [41], we found that completers had significantly better treatment outcomes than noncompleters. This highlights the importance of investigating predictors of web-CBT treatment completion.

Incidentally, we found that patients with higher BMI completed treatment more often. This is in contrast to observations by Werz et al [28]. On the basis of the current scientific knowledge, we have no reason to interpret our findings as clinically relevant.

This study reported a positive association between TA ratings and treatment completion. More specifically, the univariate models indicated that all HAQ scales (helpfulness, cooperation, and the total score), as scored by both patients and therapists, were predictors of treatment completion both at interim and post treatment. However, the multivariate model indicated that only the patients’ HAQ-total score and therapists’ HAQ-Helpfulness score were positively associated with treatment completion. This might indicate that treatment noncompletion could be reduced by improving the TA.

It should be noted that the results of this study are limited by a lack of consensus within the field of TA research concerning the definition and operationalization of TA. Across studies, a wide diversity of measures, such as the Working Alliance Inventory [67] and Therapeutic Alliance Scale [68], are used to operationalize the TA and were designed for face-to-face treatments [37]. This reduces the cross-comparability between studies. Establishing a consensus concerning the operationalization of the construct of TA in EDs and other psychotherapeutic treatments, specifically focusing on web-based treatment, would therefore be very welcome. It should also be noted that the HAQ does not provide norm scores regarding the quality of the TA, which makes it difficult to determine whether the TA is good. No clinically relevant differences in TA were determined.

Owing to the rapid development of mobile- and internet-based technology, tools and apps that are integrated into mobile devices such as smartphones are increasingly being used. The data of this study were collected from 2011 to 2013 and have already shown the importance of investing in TA because it could contribute to less psychopathology and more treatment completion. With the increased options for interactivity, it is becoming increasingly interesting to study the impact of TA in web-based treatment.

For future studies, we suggest including a more extensive population because it could lead to different results and insights. For example, male patients with ED are increasingly recognized and have unique concerns regarding disordered eating and body image [69]. The same applies to patients with anorexia nervosa. In this population, high dropout rates have been reported [70], and the strength of the TA has been shown to be associated with changes in ED symptoms [71,72].

It would also be interesting to include a face-to-face CBT condition, as this allows a comparative estimation of the effectiveness of TA on treatment outcome and treatment completion.

Finally, monitoring TA from the patient’s perspective and acting on relatively low and diminishing scores throughout the treatment process might be fruitful for clinical practice and contribute to better treatment results and completion.

### Conclusions

The results of this study showed that the strength of the TA during web-CBT for ED increased for patients who completed the program and decreased for patients who did not from both the perspectives of patients and therapists. Our study also showed that TA is predictive of ED pathology and treatment completion. In particular, patients’ confidence in their own abilities, measured using the HAQ-Helpfulness subscale, is important for predicting posttreatment ED pathology and treatment completion.

## Abbreviations

BED: binge eating disorder
BN: bulimia nervosa
CBT: cognitive behavioral therapy
DSM-IV: Diagnostic and Statistical Manual of Mental Disorders, Fourth Edition
ED: eating disorder
EDE-Q: Eating Disorder Examination Questionnaire
EDNOS: eating disorder not otherwise specified
HAQ: Helping Alliance Questionnaire
ICC: intraclass correlation coefficient
OR: odds ratio
TA: therapeutic alliance

## References

[1] Agras WS (2001). The consequences and costs of the eating disorders. Psychiatr Clin North Am.

[2] Preti A, Girolamo GD, Vilagut G, Alonso J, Graaf RD, Bruffaerts R, Demyttenaere K, Pinto-Meza A, Haro JM, Morosini P, ESEMeD-WMH Investigators (2009). The epidemiology of eating disorders in six European countries: results of the ESEMeD-WMH project. J Psychiatr Res.

[3] Ali K, Farrer L, Fassnacht DB, Gulliver A, Bauer S, Griffiths KM (2017). Perceived barriers and facilitators towards help-seeking for eating disorders: a systematic review. Int J Eat Disord.

[4] Hart LM, Granillo MT, Jorm AF, Paxton SJ (2011). Unmet need for treatment in the eating disorders: a systematic review of eating disorder specific treatment seeking among community cases. Clin Psychol Rev.

[5] J Banasiak S, J Paxton S, J Hay P (1998). Evaluating accessible treatments for bulimic eating disorders in primary care. Aust J Prim Health.

[6] Becker AE, Hadley Arrindell A, Perloe A, Fay K, Striegel-Moore RH (2010). A qualitative study of perceived social barriers to care for eating disorders: perspectives from ethnically diverse health care consumers. Int J Eat Disord.

[7] Cachelin FM, Striegel-Moore RH (2006). Help seeking and barriers to treatment in a community sample of Mexican American and European American women with eating disorders. Int J Eat Disord.

[8] Evans EJ, Hay PJ, Mond J, Paxton SJ, Quirk F, Rodgers B, Jhajj AK, Sawoniewska MA (2011). Barriers to help-seeking in young women with eating disorders: a qualitative exploration in a longitudinal community survey. Eat Disord.

[9] Schmidt U (2003). Getting technical. Eur Eat Disorders Rev.

[10] Agras WS, Fitzsimmons-Craft EE, Wilfley DE (2017). Evolution of cognitive-behavioral therapy for eating disorders. Behav Res Ther.

[11] Barakat S, Maguire S, Smith KE, Mason TB, Crosby RD, Touyz S (2019). Evaluating the role of digital intervention design in treatment outcomes and adherence to eTherapy programs for eating disorders: a systematic review and meta-analysis. Int J Eat Disord.

[12] Fitzsimmons-Craft EE, Taylor CB, Graham AK, Sadeh-Sharvit S, Balantekin KN, Eichen DM, Monterubio GE, Goel NJ, Flatt RE, Karam AM, Firebaugh M, Jacobi C, Jo B, Trockel MT, Wilfley DE (2020). Effectiveness of a digital cognitive behavior therapy-guided self-help intervention for eating disorders in college women: a cluster randomized clinical trial. JAMA Netw Open.

[13] Jenkins PE, Luck A, Violato M, Robinson C, Fairburn CG (2021). Clinical and cost-effectiveness of two ways of delivering guided self-help for people with an eating disorder: a multi-arm randomized controlled trial. Int J Eat Disord.

[14] Jensen E, Linnet J, Holmberg T, Tarp K, Nielsen J, Lichtenstein M (2020). Effectiveness of internet-based guided self-help for binge-eating disorder and characteristics of completers versus noncompleters. Int J Eat Disord.

[15] Machado PP, Rodrigues TF (2019). Treatment delivery strategies for eating disorders. Curr Opin Psychiatry.

[16] Moghimi E, Davis C, Rotondi M (2021). The efficacy of eHealth interventions for the treatment of adults diagnosed with full or subthreshold binge eating disorder: systematic review and meta-analysis. J Med Internet Res.

[17] Schuster R, Berger T, Laireiter A (2017). Computer und Psychotherapie – geht das zusammen?. Psychotherapeut.

[18] Taylor C, Graham A, Flatt R, Waldherr K, Fitzsimmons-Craft E (2021). Current state of scientific evidence on internet-based interventions for the treatment of depression, anxiety, eating disorders and substance abuse: an overview of systematic reviews and meta-analyses. Eur J Public Health.

[19] ter Huurne ED, de Haan HA, Postel MG, van der Palen J, VanDerNagel JE, DeJong CA (2015). Web-based cognitive behavioral therapy for female patients with eating disorders: randomized controlled trial. J Med Internet Res.

[20] Wyssen A, Meyer AH, Messerli-Bürgy N, Forrer F, Vanhulst P, Lalanne D, Munsch S (2021). BED-online: acceptance and efficacy of an internet-based treatment for binge-eating disorder: a randomized clinical trial including waitlist conditions. Eur Eat Disord Rev.

[21] Vollert B, Beintner I, Musiat P, Gordon G, Görlich D, Nacke B, Schmidt-Hantke J, Potterton R, Spencer L, Grant N, Schmidt U, Jacobi C (2019). Using internet-based self-help to bridge waiting time for face-to-face outpatient treatment for Bulimia Nervosa, Binge Eating Disorder and related disorders: study protocol of a randomized controlled trial. Internet Interv.

[22] Yim SH, Schmidt U (2019). Experiences of computer-based and conventional self-help interventions for eating disorders: a systematic review and meta-synthesis of qualitative research. Int J Eat Disord.

[23] Nienhuis JB, Owen J, Valentine JC, Winkeljohn Black S, Halford TC, Parazak SE, Budge S, Hilsenroth M (2018). Therapeutic alliance, empathy, and genuineness in individual adult psychotherapy: a meta-analytic review. Psychother Res.

[24] Horvath AO, Del Re AC, Flückiger C, Symonds D (2011). Alliance in individual psychotherapy. Psychotherapy (Chic).

[25] Flückiger C, Del Re AC, Wampold BE, Horvath AO (2018). The alliance in adult psychotherapy: a meta-analytic synthesis. Psychotherapy (Chic).

[26] Norcross JC, Lambert MJ (2018). Psychotherapy relationships that work III. Psychotherapy (Chic).

[27] Probst G, Berger T, Flückiger C (2019). Die allianz als prädiktor für den therapieerfolg internetbasierter interventionen bei psychischen störungen: eine korrelative metaanalyse. Verhaltenstherapie.

[28] Werz J, Voderholzer U, Tuschen-Caffier B (2022). Alliance matters: but how much? A systematic review on therapeutic alliance and outcome in patients with anorexia nervosa and bulimia nervosa. Eat Weight Disord.

[29] Puls H, Schmidt R, Hilbert A (2019). Therapist adherence and therapeutic alliance in individual cognitive-behavioural therapy for adolescent binge-eating disorder. Eur Eat Disord Rev.

[30] Graves TA, Tabri N, Thompson-Brenner H, Franko DL, Eddy KT, Bourion-Bedes S, Brown A, Constantino MJ, Flückiger C, Forsberg S, Isserlin L, Couturier J, Paulson Karlsson G, Mander J, Teufel M, Mitchell JE, Crosby RD, Prestano C, Satir DA, Simpson S, Sly R, Lacey JH, Stiles-Shields C, Tasca GA, Waller G, Zaitsoff SL, Rienecke R, Le Grange D, Thomas JJ (2017). A meta-analysis of the relation between therapeutic alliance and treatment outcome in eating disorders. Int J Eat Disord.

[31] Andersson G, Paxling B, Wiwe M, Vernmark K, Felix CB, Lundborg L, Furmark T, Cuijpers P, Carlbring P (2012). Therapeutic alliance in guided internet-delivered cognitive behavioural treatment of depression, generalized anxiety disorder and social anxiety disorder. Behav Res Ther.

[32] Andersson G, Cuijpers P, Carlbring P, Riper H, Hedman E (2014). Guided internet-based vs. face-to-face cognitive behavior therapy for psychiatric and somatic disorders: a systematic review and meta-analysis. World Psychiatry.

[33] Berger T (2017). The therapeutic alliance in internet interventions: a narrative review and suggestions for future research. Psychother Res.

[34] Clarke J, Proudfoot J, Whitton A, Birch M, Boyd M, Parker G, Manicavasagar V, Hadzi-Pavlovic D, Fogarty A (2016). Therapeutic alliance with a fully automated mobile phone and web-based intervention: secondary analysis of a randomized controlled trial. JMIR Ment Health.

[35] Simpson SG, Reid CL (2014). Therapeutic alliance in videoconferencing psychotherapy: a review. Aust J Rural Health.

[36] Steel K, Cox D, Garry H (2011). Therapeutic videoconferencing interventions for the treatment of long-term conditions. J Telemed Telecare.

[37] Sucala M, Schnur JB, Constantino MJ, Miller SJ, Brackman EH, Montgomery GH (2012). The therapeutic relationship in e-therapy for mental health: a systematic review. J Med Internet Res.

[38] Schlegl S, Bürger C, Schmidt L, Herbst N, Voderholzer U (2015). The potential of technology-based psychological interventions for anorexia and bulimia nervosa: a systematic review and recommendations for future research. J Med Internet Res.

[39] Ertelt TW, Crosby RD, Marino JM, Mitchell JE, Lancaster K, Crow SJ (2011). Therapeutic factors affecting the cognitive behavioral treatment of bulimia nervosa via telemedicine versus face-to-face delivery. Int J Eat Disord.

[40] Hamatani S, Numata N, Matsumoto K, Sutoh C, Ibuki H, Oshiro K, Tanaka M, Setsu R, Kawasaki Y, Hirano Y, Shimizu E (2019). Internet-based cognitive behavioral therapy via videoconference for patients with bulimia nervosa and binge-eating disorder: pilot prospective single-arm feasibility trial. JMIR Form Res.

[41] Ter Huurne ED, Postel MG, de Haan HA, van der Palen J, DeJong CA (2017). Treatment dropout in web-based cognitive behavioral therapy for patients with eating disorders. Psychiatry Res.

[42] Laws HB, Constantino MJ, Sayer AG, Klein DN, Kocsis JH, Manber R, Markowitz JC, Rothbaum BO, Steidtmann D, Thase ME, Arnow BA (2017). Convergence in patient-therapist therapeutic alliance ratings and its relation to outcome in chronic depression treatment. Psychother Res.

[43] Tschuschke V, Koemeda-Lutz M, von Wyl A, Crameri A, Schulthess P (2020). The impact of patients' and therapists' views of the therapeutic alliance on treatment outcome in psychotherapy. J Nerv Ment Dis.

[44] ter Huurne ED, Postel MG, de Haan HA, DeJong CA (2013). Effectiveness of a web-based treatment program using intensive therapeutic support for female patients with bulimia nervosa, binge eating disorder and eating disorders not otherwise specified: study protocol of a randomized controlled trial. BMC Psychiatry.

[45] Ter Huurne Elke D, de Haan Hein A, Postel Marloes G, DeJong Cor A J, VanDerNagel Joanne E L, van der Palen Job (2021). Long-term effectiveness of web-based cognitive behavioral therapy for patients with eating disorders. Eat Weight Disord.

[46] Sheehan DV, Lecrubier Y, Sheehan KH, Amorim P, Janavs J, Weiller E, Hergueta T, Baker R, Dunbar GC (1998). The Mini-International Neuropsychiatric Interview (M.I.N.I.): the development and validation of a structured diagnostic psychiatric interview for DSM-IV and ICD-10. J Clin Psychiatry.

[47] Overbeek I, Schruers K, Griez E, Overbeek JM (1999). Mini International Neuropsychiatric Interview: Nederlandse Versie 5.0.0, DSM-IV [Dutch Version]. Mini International Neuropsychiatric Interview: Nederlandse Versie 5.0.0, DSM-IV [Dutch Version].

[48] Brownley KA, Berkman ND, Sedway JA, Lohr KN, Bulik CM (2007). Binge eating disorder treatment: a systematic review of randomized controlled trials. Int J Eat Disord.

[49] Shapiro JR, Berkman ND, Brownley KA, Sedway JA, Lohr KN, Bulik CM (2007). Bulimia nervosa treatment: a systematic review of randomized controlled trials. Int J Eat Disord.

[50] Wilson GT, Grilo CM, Vitousek KM (2007). Psychological treatment of eating disorders. Am Psychol.

[51] Britt E, Hudson SM, Blampied NM (2004). Motivational interviewing in health settings: a review. Patient Educ Couns.

[52] Miller JH, Moyers T (2002). Motivational interviewing in substance abuse: applications for occupational medicine. Occup Med.

[53] Luborsky L, McLellan AT, Woody GE, O'Brien CP, Auerbach A (1985). Therapist success and its determinants. Arch Gen Psychiatry.

[54] De Weert-Van Oene GH, De Jong CA, Jörg F, Schrijvers GJ (1999). The Helping Alliance Questionnaire: psychometric properties in patients with substance dependence. Subst Use Misuse.

[55] (1986). The Penn helping alliance scales. The Psychotherapeutic Process A Research Handbook.

[56] Fairburn C, Beglin S (1994). Assessment of eating disorders: interview or self-report questionnaire?. Int J Eat Disord.

[57] Byrne SM, Allen KL, Lampard AM, Dove ER, Fursland A (2010). The factor structure of the eating disorder examination in clinical and community samples. Int J Eat Disord.

[58] Black CM, Wilson GT (1996). Assessment of eating disorders: interview versus questionnaire. Int J Eat Disord.

[59] (2008). Eating disorder examination Questionnaire (Edition 6.0D). Cognitive Behavior Therapy and Eating Disorders.

[60] (2013). IBM SPSS Statistics for Windows, Version 20.0.

[61] Cohen J (1988). The effect size. Statistical Power Analysis for the Behavioral Sciences.

[62] Koo TK, Li MY (2016). A guideline of selecting and reporting intraclass correlation coefficients for reliability research. J Chiropr Med.

[63] Tschuschke V, Crameri A, Koehler M, Berglar J, Muth K, Staczan P, Von Wyl A, Schulthess P, Koemeda-Lutz M (2015). The role of therapists' treatment adherence, professional experience, therapeutic alliance, and clients' severity of psychological problems: prediction of treatment outcome in eight different psychotherapy approaches. Preliminary results of a naturalistic study. Psychother Res.

[64] Leibert T, Archer J, Munson J, York G (2006). An exploratory study of client perceptions of internet counseling and the therapeutic alliance. J Mental Health Counsel.

[65] Dölemeyer R, Klinitzke G, Steinig J, Wagner B, Kersting A (2013). [Working alliance in internet-based therapy for binge eating disorder]. Psychiatr Prax.

[66] Kelders SM, Kok RN, Ossebaard HC, Van Gemert-Pijnen JE (2012). Persuasive system design does matter: a systematic review of adherence to web-based interventions. J Med Internet Res.

[67] Horvath A, Greenberg L (1986). The development of the working alliance inventory. The Psychotherapeutic Process: A Research Handbook.

[68] Bickman L, Vides de Andrade AR, Warren Lambert E, Doucette A, Sapyta J, Suzanne Boyd A, Rumberger DT, Moore-Kurnot J, McDonough LC, Rauktis MB (2004). Youth therapeutic alliance in intensive treatment settings. J Behav Health Services Res.

[69] Nagata JM, Ganson KT, Murray SB (2020). Eating disorders in adolescent boys and young men: an update. Curr Opin Pediatr.

[70] Lock J, Brandt H, Woodside B, Agras S, Halmi WK, Johnson C, Kaye W, Wilfley D (2012). Challenges in conducting a multi-site randomized clinical trial comparing treatments for adolescent anorexia nervosa. Int J Eat Disord.

[71] Isserlin L, Couturier J (2012). Therapeutic alliance and family-based treatment for adolescents with anorexia nervosa. Psychotherapy (Chic).

[72] Pereira T, Lock J, Oggins J (2006). Role of therapeutic alliance in family therapy for adolescent anorexia nervosa. Int J Eat Disord.

